# Safety of UFT/LV and S-1 as adjuvant therapy for stage III colon cancer in phase III trial: ACTS-CC trial

**DOI:** 10.1038/bjc.2012.86

**Published:** 2012-03-13

**Authors:** I Mochizuki, H Takiuchi, K Ikejiri, Y Nakamoto, Y Kinugasa, A Takagane, T Endo, H Shinozaki, Y Takii, Y Takahashi, H Mochizuki, K Kotake, S Kameoka, K Takahashi, T Watanabe, M Watanabe, N Boku, N Tomita, Y Matsubara, K Sugihara

**Affiliations:** 1 Department of Gastroenterological Surgery, Iwate Central Prefectural Hospital, 1-4-1 Ueda, Morioka, Iwate 020-0066, Japan; 2 Cancer Chemotherapy Center, Osaka Medical College, 2-7 Daigaku-machi, Takatsuki, Osaka 569-8686, Japan; 3 Department of Surgery, Gastrointestinal Center, National Hospital Organization Kyushu Medical Center, 1-8-1 Jigyohama, Chuo-ku, Fukuoka 810-8563, Japan; 4 Department of Surgery, Kobe City Medical Center West Hospital, 1-2-4 Nagata-ku, Kobe, Hyogo 653-0013, Japan; 5 Division of Colon and Rectal Surgery, Shizuoka Cancer Center Hospital, 1007 Shimonagakubo, Nagaizumi-cho, Sunto-gun, Shizuoka 411-8777, Japan; 6 Department of Surgery, Hakodate Goryoukaku Hospital, 38-3 Goryoukaku-cho, Hakodate, Hokkaido 040-8611, Japan; 7 Department of Coloproctological Surgery, Japanese Red Cross Medical Center, 4-1-22 Hiroo, Shibuya-ku, Tokyo 150-8935, Japan; 8 Department of Surgery, Saiseikai Utsunomiya Hospital, 911-1 Takebayashi, Utsunomiya, Tochigi 321-0974, Japan; 9 Department of Surgery, Niigata Cancer Center Hospital, 2-15-3, Kawagishi-cho, Chuo-ku, Niigata, Niigata 951-8566, Japan; 10 Department of Surgery, Ogaki Municipal Hospital, 4-86 Minaminokawa-cho, Ogaki, Gifu 503-8502, Japan; 11 Department of Surgery, National Defense Medical College, 3-2 Namiki, Tokorozawa, Saitama 359-8513, Japan; 12 Department of Surgery, Tochigi Cancer Center, 4-9-13 Yonan, Utsunomiya, Tochigi 320-0834, Japan; 13 Department of Surgery II, Tokyo Women's Medical University, 8-1 Kawada-cho, Shinjyuku-ku, Tokyo 162-8666, Japan; 14 Department of Surgery, Cancer and Infectious Diseases Center Komagome Hospital, 18-22, Honkomagome 3-chome, Bunkyo-ku, Tokyo 113-8677, Japan; 15 Department of Surgery, Teikyo University, 2-11-1 Kaga, Itabashi-ku, Tokyo 173-8605, Japan; 16 Department of Surgery, Kitasato University School of Medicine, 1-15-1 Kitasato, Minami-ku, Sagamihara, Kanagawa 252-0375, Japan; 17 Department of Clinical Oncology, St Marianna University School of Medicine, 2-16-1 Sugao, Miyamae-ku, Kawasaki, Kanagawa 216-8511, Japan; 18 Department of Surgery, Hyogo College of Medicine, 1-1 Mukogawa-cho, Nishinomiya, Hyogo 663-8501, Japan; 19 Department of Data Management and Analysis, Translational Research Informatics Center, 1-5-4 Minatojima-minamimachi, Chuo-ku, Kobe, Hyogo 650-0047, Japan; 20 Department of Surgical Oncology, Tokyo Medical and Dental University, Graduate School, 1-5-45 Yushima, Bunkyo-ku, Tokyo 113-8519, Japan

**Keywords:** colon cancer, adjuvant chemotherapy, phase III, S-1, UFT

## Abstract

**Background:**

The Adjuvant Chemotherapy Trial of TS-1 for Colon Cancer (ACTS-CC) is a phase III trial designed to validate the non-inferiority of S-1 to UFT/leucovorin (LV) as postoperative adjuvant chemotherapy for stage III colon cancer. We report the results of a planned safety analysis.

**Methods:**

Patients aged 20–80 years with curatively resected stage III colon cancer were randomly assigned to receive UFT/LV (UFT, 300 mg m^−2^ per day as tegafur; LV, 75 mg per day on days 1–28, every 35 days, 5 courses) or S-1 (80, 100, or 120 mg per day on days 1–28, every 42 days, 4 courses). Treatment status and safety were evaluated.

**Results:**

Of 1535 enrolled patients, a total of 1504 (756 allocated to S-1 and 748 to UFT/LV) were analysed. The completion rate of protocol treatment was 77% in the S-1 group and 73% in the UFT/LV group. The overall incidence of adverse events (AEs) were 80% in S-1 and 74% in UFT/LV. Stomatitis, anorexia, hyperpigmentation, and haematological toxicities were common in S-1, whereas increased alanine aminotransferase and aspartate aminotransferase were common in UFT/LV. The incidences of ⩾grade 3 AEs were 16% and 14%, respectively.

**Conclusion:**

Although AE profiles differed between the groups, feasibility of the protocol treatment was good. Both S-1 and UFT/LV could be safely used as adjuvant chemotherapy.

Colorectal cancer (CRC) was the second most common cancer in Japan, affecting over 100 000 individuals (Cancer statistics in Japan, 2010). The Japanese Society for Cancer of the Colon and Rectum (JSCCR) reported that recurrence rates were 3.7% for stage I disease, 13.3% for stage II, and 30.8% for stage III (Kobayashi *et al*, 2007). Postoperative adjuvant chemotherapy for patients with stage III CRC is now internationally accepted as a standard care to improve outcomes. In the mid-1990s, 6 months of intravenous (i.v.) therapy with 5-fluorouracil (5-FU)/leucovorin (LV) was established to be standard adjuvant chemotherapy for colon cancer. Subsequently, the benefits of adding oxaliplatin to 5-FU/LV were evaluated. At present, 5-FU/LV combined with oxaliplatin is regarded as the standard adjuvant chemotherapy for stage III colon cancer in western countries (Labianca *et al*, 2010; National comprehensive cancer network (NCCN), 2011).

The JSCCR Guidelines 2010 for the Treatment of Colorectal Cancer (Japanese Society for Cancer of the Colon and Rectum, 2010) recommend four regimens as adjuvant therapy for stage III CRC: i.v. 5-FU/LV, UFT/LV, capecitabine, and FOLFOX (5-FU/LV plus oxaliplatin). However, large population database demonstrated that outcomes differ among subgroups of patients with stage III disease (Gunderson *et al*, 2010). Consequently, in Japan, considering expected benefits and possible risks of increased toxicity, a consensus has not been reached as to whether adjuvant regimens containing oxaliplatin should be given to all patients with stage III disease. Actually, in Japan, several oral 5-FU derivatives are available, and oral 5-FU agents have been preferred because of their convenience. About 80% of CRC patients received adjuvant chemotherapy using oral 5-FU agents. UFT/LV is one of the most widely used regimens for adjuvant chemotherapy of stage III CRC in Japan.

UFT (Taiho Pharmaceutical Co., Ltd, Tokyo, Japan) is an oral 5-FU derivative that combines tegafur with uracil in a molar ratio of 1 : 4. Tegafur is a prodrug of 5-FU, and uracil competitively inhibits the degradation of 5-FU by dihydropyrimidine dehydrogenase (DPD). Concomitant use of the oral folic acid derivative LV with UFT promotes stabilising the ternary complex and augmenting the inhibition of thymidylate synthase (TS) by 5-FU. The National Surgical Adjuvant Breast and Bowel Project (NSABP) C-06 trial, which enrolled 1608 patients with stage II or III colon cancer in the United States, demonstrated non-inferiority of UFT/LV to i.v. 5-FU/LV in terms of efficacy and safety (Lembersky *et al*, 2006), and demonstrated better convenience of UFT/LV (Kopec *et al*, 2007).

S-1 (TS-1; Taiho Pharmaceutical Co., Ltd) is another oral 5-FU derivative available for CRC in Japan. It combines tegafur, gimeracil, and oteracil, in a molar ratio of 1 : 0.4 : 1 (Shirasaka *et al*, 1996). Gimeracil, a DPD inhibitor, is about 200-fold more potent than uracil. Oteracil inhibits the conversion of 5-FU to active metabolites in the gastrointestinal tract, resulting in reduction of gastrointestinal toxicity of 5-FU. The phase II trial of monotherapy with S-1 for metastatic CRC showed response rates about 35%, which were higher than that of UFT/LV (Ohtsu *et al*, 2000). In a large phase III study in patients with stage II and III gastric cancer (Adjuvant Chemotherapy Trial of TS-1 for Gastric Cancer (ACTS-GC) trial), 1 year of postoperative adjuvant chemotherapy with S-1 compared with surgery alone disclosed significantly prolonged relapse-free survival and overall survival (Sakuramoto *et al*, 2007). S-1 is now widely used as the standard adjuvant chemotherapy for GC. However, the efficacy of S-1 as adjuvant chemotherapy on CRC has not been established.

S-1 has several advantages, including slightly higher anti-tumour activity, low costs, and easy administration, that is, twice daily after meals (UFT/LV is given three times daily, more than 1 h after or before meals). In addition, because of differences in the mechanisms of action, S-1 may be useful in a different subset of patients and have a distinct toxicity profile from that of UFT/LV. S-1 may thus become a new, more convenient option of adjuvant regimen.

We designed a study named ACTS-CC (ACTS for Colon Cancer) to verify the non-inferiority of S-1 to UFT/LV, and thereby confirm the usefulness of adjuvant chemotherapy with S-1 for stage III CRC (ClinicalTrials.gov: no. NCT00660894). The primary endpoint is disease-free survival rate at 3 years after finishing enrolment. Enrolment started in April 2008 and was completed in June 2009. Final conclusions regarding the therapeutic usefulness of these regimens will be open in 2012. But, safety data of UFT/LV and S-1 from large trials with CRC is still unclear, although they are now widely used clinically in Japan. We therefore report the results of a planned interim analysis limited to the safety data in this study, to contribute to the safer use of these regimens in clinical practice.

## Materials and methods

### Enrolment and assignment

This study was conducted in accordance with the ‘Declaration of Helsinki’ and the ‘Ethical Guidelines for Clinical Research’, and was approved by the Institutional Review Boards of each participating institute. Written informed consent was obtained from all patients before enrolment.

Eligible patients were centrally registered by using a Web enrolment system. The main eligible criteria were as follows: aged 20–80 years, histologically confirmed stage III colon adenocarcinoma after curative surgery, starting chemotherapy within 8 weeks after surgery, performance status of 0–1, adequate oral intake, and preserved major organ functions.

### Randomisation and masking

After confirming eligibility, enrolled patients were randomly assigned to receive either UFT/LV or S-1 at the central registration centre by a computer programme, by use of a minimisation method with stratification by lymph node metastasis (N1 or N2) and institution. Assignment of patients was concealed from the investigator. Treatment assignment was not masked from the investigators or patients.

### Protocol treatment

In the UFT/LV arm, UFT was given at a dose of 300 mg m^−2^ per day as tegafur in three divided doses (every 8 h) more than 1 h after or before meals. A quantity of 75 mg per body per day of LV was given in three divided doses simultaneously with UFT. These drugs were orally administered for 28 consecutive days, followed by a 7-day rest. This 5-week treatment comprised one course. A total of five courses (25 weeks) were delivered.

In the S-1 arm, S-1 was orally given at a dose according to body surface area (BSA; 40 mg with BSA <1.25 m^2^; 50 mg with BSA 1.25–1.5 m^2^; 60 mg with BSA >1.5 m^2^) twice daily after meals for 28 consecutive days, followed by a 14-day rest. This 6-week treatment comprised one course. A total of four courses (24 weeks) were delivered.

Assigned treatment was started within 8 weeks after surgery. During protocol treatment, clinical findings and laboratory values were evaluated every 2 to 3 weeks (evaluations at the time of starting each course were mandatory). Protocol treatment in each course was started and continued when the patients fulfilled the criteria included: leukocytes ⩾3000/mm^3^, platelets ⩾100 000/mm^3^, haemoglobin ⩾9.0 g dl^−1^, aspartate aminotransferase (AST) and alanine aminotransferase (ALT) ⩽100 IU l^−1^, total bilirubin ⩽2.0 mg dl^−1^, creatinine <1.5 mg dl^−1^, no greater than grade 1 anorexia, nausea, vomiting, and diarrhoea. If the criteria for starting/continuing treatment are not met, treatment was postponed or temporarily suspended until adverse events (AEs) had become to meet the criteria. Depending upon the severity of AEs, the dose of UFT or S-1 was reduced in accordance with the protocol when the treatment was resumed. Once the dose had been reduced, it was not to be increased at a later time. In the UFT/LV group, the dose of LV was not modified.

Protocol treatment was discontinued in the cases included: recurrence or other malignancies developed, treatment failed to be resumed within 14 days after being postponed or temporarily suspended (the pre-defined drug rest for each group is not included), further dose reduction was necessary because of AEs, and so on, even after the specified dose was reduced by two levels or to minimal dose level, the physician judged that the protocol treatment was difficult to continue, the patient requested discontinuation of protocol treatment, and the patients withdrew informed consent.

### Data collection

#### Treatment status

Physicians reported the treatment status (i.e., the number of days of administration in each course) by a Web-based case report system.

The drug compliance for each course was defined as the ratio of the actually taken dose to the prescribed dose, and was classified to the following four categories: (1) ⩾90% taken, (2) ⩾75% to <90% taken, (3) ⩾50% to <75% taken, and (4) <50% taken.

Using reported information in the case report form, taken dose per course was calculated for each patient as follows: (prescribed daily dose) × (number of days of administration) × (oral drug compliance for each course). Relative dose intensity for each patient was defined as the ratio of cumulative taken dose during the entire treatment period to scheduled total dose per protocol.

Completion rate of protocol treatment was defined as the ratio of the number of patients who completed four courses of S-1 treatment or five courses of UFT/LV treatment to the number of patients included in the safety analysis set of each group.

#### Safety profile

The types and severities of AEs from the start of protocol treatment to 30 days after the last administration were evaluated according to the National Cancer Institute Common Terminology Criteria for Adverse Events version 3.0 (National Cancer Institute, Bethesda, MD, USA). The most severe grade of each AE during each course was reported. The following AEs were required to report as ‘priority survey items’: leukocytes, haemoglobin, platelets, total bilirubin, AST, ALT, creatinine, stomatitis, anorexia, nausea, vomiting, diarrhoea, rash/desquamation, hyperpigmentation, and fatigue.

### Statistical analysis

Data were analysed using SAS (Statistical Analysis System) version 9.1.2 (SAS Institute Inc., Cary, NC, USA). Descriptive statistics such as means, s.d., and medians were calculated. The incidences of categorised discrete values were expressed as percentages for each group.

## Results

### Patients' characteristics

**Figure 1:**
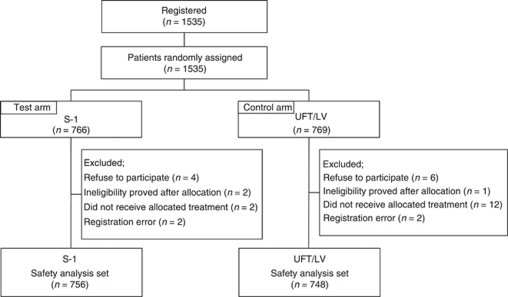
CONSORT diagram.

From April 2008 through June 2009, a total of 1535 patients were enrolled from 358 hospitals in Japan. After excluding 31 patients because of the reasons shown in Figure 1, 1504 were included in the safety analysis (756 in the S-1 group and 748 in the UFT/LV group). The data were cut off on 11 August 2010. The characteristics of the 1504 patients are shown in Table 1.

### Treatment status

Completion rate of protocol treatment was 76.5% in the S-1 group and 73.4% in the UFT/LV group (Table 2). Discontinuation of protocol treatment was most common during course 1 and then decreased with courses. Among the 377 patients with discontinuation of the protocol treatments, 138 (77.5% of 178 discontinuation cases) in the S-1 group and 133 (66.8% of 199 discontinuation cases) in the UFT/LV group did within the first two courses (Table 2). Treatment discontinuation because of AEs was observed in 132 patients in the S-1 group and in 134 in the UFT/LV group. Among these patients, treatment was discontinued in 54 patients by the AEs listed in the discontinuation criteria of the protocol, in 34 by physician's decision for other than protocol criteria, and in 44 by patient's refusal related to AEs of the S-1 group, and in 67, 34, 41 of the UFT/LV group, respectively.

**Figure 2:**
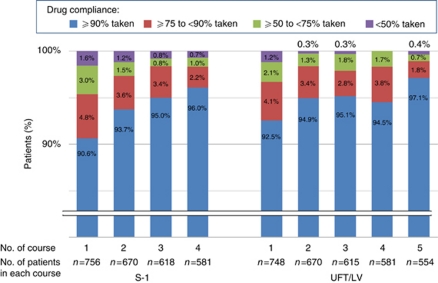
Drug compliance in each course. Each shaded region represents the percentage of patients receiving the indicated proportion of the scheduled dose per protocol in a given course. Abbreviation: LV=leucovorin.

As for drug compliance, more than 90% of patients in both groups were reported to take ‘⩾90%’ of prescribed dose for each course (Figure 2). The mean of relative dose intensity, including discontinuation cases, was 76.5% in the S-1 group and 76.0% in the UFT/LV group; the median was 95% in both groups.

### Safety profile

A total of 605 patients (80.8%) in the S-1 group and 551 (73.7%) in the UFT/LV group experienced AEs (any grades). In all, 121 patients (16.0%) in the S-1 group and 108 (14.4%) in the UFT/LV group experienced ⩾grade 3 AEs. The incidences of AEs pre-specified as ‘priority survey items’ are shown in Table 3. The common AEs in any grades were anorexia, diarrhoea, fatigue, anaemia, and hyperbilirubinemia. Stomatitis, anorexia, rash/desquamation, hyperpigmentation, leukopenia, anaemia, and thrombocytopenia were more frequent in the S-1 group. Increased ALT and AST levels were more frequent in the UFT/LV group.

In the UFT/LV group, 5 patients (0.7%) experienced grade 4 increased ALT levels, and 3 (0.4%) had grade 4 increased AST levels (some overlap). One patient in the S-1 group had grade 4 increased AST level. All these events occurred during course 1.

Grade 4 haematological toxicities were as follows: anaemia in one patient, leukocytopenia in two, neutropenia in one in the S-1 group (some overlap), and anaemia in one patient in the UFT/LV group. Grade 3 neutropenia was developed in 10 patients (1.3%) in the S-1 group and 2 (0.3%) in the UFT/LV group.

The other common AEs in any grades were taste alteration (4.0% in the S-1 group and 3.2% in the UFT/LV group) and eye-related symptoms, including tearing, keratitis, and conjunctivitis (3.8% in the S-1 group and 0.4% in the UFT/LV group).

There were two deaths in the UFT/LV group, which was not ruled out to be related to the protocol treatment. One patient had diarrhoea leading to dehydration, metabolic acidosis, and acute respiratory distress syndrome during the first course. In the other patient, aspiration pneumonia associated with postoperative bowel obstruction, which developed during course 5, lead to respiratory failure.

## Discussion

This paper reported the results of an interim analysis of safety data obtained from the phase III study of 1504 patients with stage III colon cancer, who received postoperative adjuvant chemotherapy with UFT/LV or S-1.

The overall incidence of AEs (any grades) was 80.0% in the S-1 group and 73.7% in the UFT/LV group, and that of ⩾ grade 3 AEs were 16.0% and 14.4%, respectively. In short, about 80% of AEs were mild or moderate AEs such as grade 1 to 2. The completion rate of protocol treatment was favourable (76.5% in the S-1 group and 73.4% in the UFT/LV group), and treatment was discontinued in some patients during the early courses in both groups. Careful watch in early courses, adequate supportive care, and temporary suspension is important to complete the adjuvant chemotherapy with UFT/LV or S-1.

The present study is the first large trial of adjuvant chemotherapy with S-1 in patients with CRC. As compared, AEs of the S-1 group in this study with those of the ACTS-GC trial in which 1-year S-1 was used for adjuvant chemotherapy in GC (Sakuramoto *et al*, 2007), AE profiles in both trials were similar; the common AEs were anaemia, anorexia, diarrhoea, fatigue, and hyperpigmentation. However, the overall incidence of AEs was higher in the ACTS-GC trial. It may be because of the longer treatment duration of S-1 in the ACTS-GC trial. The proportion of patients who were in treatment at 6 months was similar: 77.9% in the ACTS-GC trial and 76.5% in this study.

On the other hand, potential racial differences of the tolerability for fluoropyrimidines had been reported (Haller *et al*, 2008). When the pharmacokinetics and pharmacodynamics of S-1 were compared between Caucasian and East Asian patients with solid malignancy including CRC, grade 3–4 gastrointestinal toxicities were more common in Caucasians than Asians, although exposure to 5-FU concentration was similar in both groups (Chuah *et al*, 2011).

In the NSABP C-06 trial (Lembersky *et al*, 2006), which was conducted in the United States, AEs in 774 patients who received UFT/LV was observed in 93.5% (⩾ grade 3, in 38.2%). Gastrointestinal toxicity (i.e., diarrhoea, nausea, and vomiting) was considerably less developed in this study, whereas the incidence of haematological toxicity was similar in both studies (Table 4). The difference of AE profiles between Japan and the United States of the bridging study of UFT/LV for unresectable CRC showed similar tendency (Shirao *et al*, 2004).

The UFT/LV treatment sometimes causes liver dysfunction (i.e., increased AST, ALT levels, and hyperbilirubinemia). In this study, five patients (0.7%) in the UFT/LV group had grade 4 liver dysfunction; all cases developed during course 1. The survey performed by the pharmaceutical company reported the similar observations of liver dysfunction caused by UFT, with the highest incidence within 2 months after start of treatment. Therefore, patients treated with UFT/LV are better to be watched carefully about liver dysfunction, and liver function is recommended to be regularly evaluated in early period in treatment.

Because of the different mechanisms of action between S-1 and UFT/LV, AE profiles were expected to differ between two groups. The common AEs were stomatitis, anorexia, rash/desquamation, hyperpigmentation, leukopenia, anaemia, and thrombocytopenia in the S-1 group, and increased ALT and AST levels in the UFT/LV group. Derivatives of 5-FU have been reported to cause keratoconjunctival epithelial disorders due to impaired DNA synthesis, which lead to secondary tear-duct occlusion accompanied by lacrimation (Hassan *et al*, 1998). This study disclosed that the incidence of eye-related symptoms differs between S-1 and UFT/LV. This study is designed to investigate mRNA expression levels and DNA copy numbers of 5-FU-related enzymes, and to clarify relationship between AEs profiles and the results of molecular study. When final results will be open, causes of different profiles of AEs will be disclosed.

The profiles and severity of AEs in this study were not worse than the reported AEs with other regimens of adjuvant chemotherapy (Table 4), and were acceptable. Hand–foot syndrome (HFS) was more common in capecitabine (Twelves *et al*, 2005), whereas ⩾grade 3 HFS in this study was 1.3% in the S-1 group and 0.9% in the UFT/LV group. Haematological toxicities were more common in the regimens containing oxaliplatin (André *et al*, 2004). As mentioned above, gastrointestinal toxicities were fewer in this study, possibly because of racial differences.

In conclusion, the present analysis showed that the AE profiles differed between UFT/LV and S-1, whereas the incidence of ⩾grade 3 AEs was low in both groups. The high completion rate of the protocol treatment with good drug compliance may indicate both regimens are acceptable treatment as adjuvant chemotherapy for CRC.

## Figures and Tables

**Table 1:** Patients' characteristics

		**S-1**		**UFT/LV**	
		***n*=756**	**(%)**	***n*=748**	**(%)**
Age (years)	Median (range)	66 (23–80)	—	65 (32–80)	—
	⩾70 years	279	(36.9)	252	(33.7)
					
Gender	Male	411	(54.4)	397	(53.1)
	Female	345	(45.6)	351	(46.9)
					
PS (ECOG)	0	720	(95.2)	716	(95.7)
	1	36	(4.8)	32	(4.3)
					
Tumour location	Right colon (C, A, T)	324	(42.9)	262	(35.0)
	Left colon (D, S)	277	(36.6)	309	(41.3)
	Rectosigmoid	155	(20.5)	177	(23.7)
					
Depth of tumour invasion	T1	40	(5.3)	46	(6.1)
(TNM 7th)	T2	76	(10.1)	77	(10.3)
	T3	428	(56.6)	425	(56.8)
	T4	212	(28.0)	200	(26.8)
					
Extent of LN dissection^a^	D1	5	(0.7)	5	(0.7)
	D2	142	(18.8)	150	(20.1)
	D3	609	(80.6)	593	(79.3)
					
No. of LN examined	Median (range)	18 (1–78)		16 (1-78)	
	<12	181	(24.1)	206	(27.5)
	⩾12	575	(75.9)	542	(72.5)
					
LN metastasis	N1a	330	(43.7)	325	(43.4)
(TNM 7th)	N1b	265	(35.1)	263	(35.2)
	N2a	116	(15.3)	113	(15.1)
	N2b	45	(6.0)	47	(6.3)
					
Stage	IIIA	105	(13.9)	118	(15.8)
(TNM 7th)	IIIB	550	(72.8)	516	(69.0)
	IIIC	101	(13.4)	114	(15.2)

Abbreviations: ECOG=The Eastern Cooperative Oncology Group; LN=lymph node; LV=leucovorin; PS=performance status.

D1: complete dissection of pericolic/perirectal lymph nodes

D2: complete dissection of pericolic/perirectal and intermediate lymph nodes

D3: complete dissection of all regional lymph nodes.

a Extent of lymph node dissection according to Japanese Classification of Colorectal Carcinoma.

**Table 2:** Discontinuation and completion of protocol treatment

	**S-1**		**UFT/LV**	
	***n*=756**	**(%)**	***n*=748**	**(%)**
No. of patients completed the protocol treatment	578	(76.5)	549	(73.4)
				
*No. of patients with discontinuation*	178	(23.5)	199	(26.6)
During course 1	86	(11.4)	78	(10.4)
During course 2	52	(6.9)	55	(7.4)
During course 3	37	(4.9)	34	(4.5)
During course 4	3	(0.4)	27	(3.6)
During course 5	—	—	5	(0.7)

Abbreviation: LV=leucovorin.

**Table 3:** Incidence of AEs for entire treatment period (worst grade)

	**S-1**				**UFT/LV**			
	***n*=756**				***n*=748**			
	**Any grades**		**⩾Grade 3**		**Any grades**		**⩾Grade 3**	
**Events**	***n***	**(%)**	***n***	**(%)**	***n***	**(%)**	***n***	**(%)**
*Clinical findings*								
Stomatitis	146	(19.3)	9	(1.2)	103	(13.8)	3	(0.4)
Anorexia	242	(32.0)	37	(4.9)	187	(25.0)	26	(3.5)
Nausea	166	(22.0)	12	(1.6)	142	(19.0)	9	(1.2)
Vomiting	48	(6.3)	6	(0.8)	58	(7.8)	6	(0.8)
Diarrhoea	177	(23.4)	33	(4.4)	178	(23.8)	41	(5.5)
Rash/Desquamation	114	(15.1)	2	(0.3)	75	(10.0)	4	(0.5)
Hyperpigmentation	201	(26.6)	—	—	95	(12.7)	—	—
Fatigue	208	(27.5)	18	(2.4)	186	(24.9)	11	(1.5)
								
*Laboratory findings*								
Leukocytes	136	(18.0)	5	(0.7)	93	(12.4)	3	(0.4)
Haemoglobin	246	(32.5)	7	(0.9)	199	(26.6)	1	(0.1)
Platelets	96	(12.7)	1	(0.1)	55	(7.4)	3	(0.4)
Total bilirubin	195	(25.8)	9	(1.2)	173	(23.1)	11	(1.5)
AST	114	(15.1)	6	(0.8)	152	(20.3)	16	(2.1)
ALT	100	(13.2)	8	(1.1)	160	(21.4)	25	(3.3)
Creatinine	36	(4.8)	0	(0)	34	(4.5)	4	(0.5)

Abbreviations: AEs=adverse events; ALT=alanine aminotransferase; AST=aspartate aminotransferase; LV=leucovorin.

**Table 4:** Reported incidence of AEs with other regimens

	**Lembersky *et al* (2006)**				**Twelves *et al* (2005)**		**André *et al* (2004)**	
	**UFT/LV (*n*=774)**		**i.v. 5-FU/LV**^a^ **(*n*=759)**		**Capecitabine (*n*=995)**		**FOLFOX4 (*n*=1108)**	
**Events**	**Any grades (%)**	**⩾Grade 3 (%)**	**Any grades (%)**	**⩾Grade 3 (%)**	**Any grades (%)**	**⩾Grade 3 (%)**	**Any grades (%)**	**⩾Grade 3 (%)**
*Clinical findings*								
Stomatitis	26	1.3	24	0.5	22	2	42	3
Nausea	54	7	65	7	36	3	74	5
Vomiting	28	4	31	7			47	6
Diarrhoea	75	29	79	29	46	11	56	11
Skin disorders	22^b^	1.3^b^	20^b^	1.1^b^	—	—	32^b^	2^b^
HFS	—	0.7	—	0.2	60	17	—	—
Paraesthesia	—	—	—	—	—	—	92	12
								
*Laboratory findings*								
Leukocytes	17	0	22	0.7	<10	—	—	—
Granulocytes	20	1.3	27	1.3	32	2	79	41
Haemoglobin	—	—	—	—	<10	—	76	0.8
Platelets	—	—	—	—	<10	—	77	1.7
Total bilirubin	7	0.3	4	—	50	20	—	—

Abbreviations: AEs=adverse events; FOLFOX=5-FU/LV plus oxaliplatin; HFS=hand–foot syndrome; i.v.=intravenous; LV=leucovorin.

a Treatment schedule reported from the Roswell Park Memorial Institute.

b Including HFS.
