# Supplementary material for: Safety of UFT/LV and S-1 as adjuvant therapy for stage III colon cancer in phase III trial: ACTS-CC trial
Source: Br J Cancer. 2012 Mar 13;106(7):1268–73. doi: 10.1038/bjc.2012.86 (PMC3314794; doi:10.1038/bjc.2012.86)
Supplement: Supplementary Information [file bjc201286x1.doc]

**Supplementary Information (participating institutes).**

**ACTS-CC Study Group:**

Hakodate Goryoukaku Hospital (Hakodate), Hakodate Municipal Hospital (Hakodate), Otaru-Ekisaikai Hospital (Otaru), Muroran City General Hospital (Muroran), Hokkaido P.W.F.A.C Sapporo-Kosei General Hospital (Sapporo), Sapporo Medical University (Sapporo), Sapporo City General Hospital (Sapporo), Sapporo Doto Hospital (Sapporo), Takikawa Municipal Hospital (Takikawa), Fukagawa Municipal Hospital (Fukagawa), Hokkaido P.W.F.A.C Asahikawa-Kosei General Hospital (Asahikawa), Asahikawa Medical University (Asahikawa), Kobayashi Hospital (Kitami), Hokkaido P.W.F.A.C Engaru-Kosei General Hospital (Monbetsu), Hirosaki University School of Medicine & Hospital (Hirosaki), Iwate Prefectural Central Hospital (Morioka), Tohoku University school (Sendai), Tohoku Rosai Hospital (Sendai), Miyagi Cancer Center (Natori), Sendai Open Hospital (Sendai), National Hospital Organization Sendai Medical Center (Sendai), Osaki Citizen Hospital (Osaki), Akita City Hospital (Akita), Akita University Hospital (Akita), Hiraka General Hospital (Yokote), Yamagata University (Yamagata), Fukushima Medical University (Fukushima), Iwaki Kyouritsu Hospital (Iwaki), Tsuchiura Kyodo General Hospital (Tsuchiura), Tsukuba Medical Center (Tsukuba), University of Tsukuba (Tsukuba), Mito Red Cross Hospital (Mito), National Hospital Organization Mito Medical Center (Higashiibaraki), Hitachi General Hospital (Hitachi), Tochigi Cancer Center (Utsunomiya), Tochigi National Hospital (Utsunomiya), Dokkyo Medical University Hospital (Shimotsuga), Saiseikai Utsunomiya Hospital (Utsunomiya), Kamituga General Hospital (Kanuma), Ohtawara Red Cross Hospital (Ohtawara), Ashikaga Red Cross Hospital (Ashikaga), Maebashi Red Cross Hospital (Maebashi), Saiseikai Maebashi Hospital (Maebashi), Gunma University Hospital (Maebashi), Gunma University Hospital,Department of Thoracic and Visceral Organ Surgery (Maebashi), Isesaki Municipal Hospital (Isesaki), Gunma Prefectural Cancer Center (Ota), Kiryu Kosei General Hospital (Kiryu), Haramachi Red Cross Hospital (Agatsuma), Saiseikai Kawaguchi General Hospital (Kawaguchi), Saitama City Hospital (Saitama), Dokkyo Medical University Koshigaya Hospital (Koshigaya), Shuwa General Hospital (Kasukabe), Hasuda Hospital (Hasuda), Saitama Medical Center (Kawagoe), Saitama Medical University International Medical Center (Hidaka), National Defense Medical College (Tokorozawa), Saitama Cancer Center (Kitaadachi), Kitasato University Kitasato Institute Medical Center Hospital (Kitamoto), Fukaya Red Cross Hospital (Fukaya), Chiba University Hospital (Chiba), Chiba Cancer Center (Chiba), Nippon Medical School Chiba Hokusoh Hospital (Inzai), Matsudo City Hospital (Matsudo), Juntendo University Urayasu Hospital (Urayasu), Toho University Sakura Medical Center (Sakura), Asahi General Hospital (Asahi), Teikyo University Chiba Medical Center (Ichihara), St. Luke's International Hospital (Tokyo), Jikei University School of Medicine (Tokyo), International University of Health and Werfare Mita Hospital (Tokyo), Tokyo Medical And Dental University, Granduate School (Tokyo), Nippon Medical School Hospital (Tokyo), Department of Surgery, Cancer and Infectiouas diseases Center Komagome Hospital (Tokyo), Tokyo-Kita Social Insurance Hospital (Tokyo), Showa University Toyosu Hospital (Tokyo), NTT Medical Center Tokyo (Tokyo), Toho University Omori Medical Center (Tokyo), Omori Red Cross Hospital (Tokyo), Japanese Red Cross Medical Center (Tokyo), National Hospital Organization Tokyo Medical Center (Tokyo), Toho University Ohashi Medical Center (Tokyo), Tokyo Medical University Hospital (Tokyo), Keio University Hospital (Tokyo), National Center for Global Health and Medicine (Tokyo), Tokyo Women's Medical University (Tokyo), Tokyo Women's Medical University Department of Surgery II (Tokyo), Kawakita General Hospital (Tokyo), Social Insurance Chuo General Hospital (Tokyo), Tokyo Metropolitan Ohtsuka Hospital (Tokyo), Tokyo Metropolitan Health and Medical Corporation Toshima Hospital (Tokyo), Teikyo University (Tokyo), Itabashi Chuo Medical Center (Tokyo), Nerima General Hospital (Tokyo), Musashino Red Cross Hospital (Musashino), Tokyo Metropolitan Tama Medical Center (Fuchu), Showa General Hospital (Kodaira), Tokyo Medical University Hachioji Medical Center (Hachioji), Ome Municipal General Hospital (Ome), Social Medical Corporation Yamatokai Foundation Higashiyamato (Higashiyamato), Kawasaki Municipal Hospital (Kawasaki), Kanto Rosai Hospital (Kawasaki), Nippon Medical School Musashi Kosugi Hospital (Kawasaki), Kawasaki Saiwai Hospital (Kawasaki), Kawasaki Municipal Tama Hospital (Kawasaki), St. Marianna University School of Medicine (Kawasaki), Yokohama Rosai Hospital (Yokohama), Showa University Northern Yokohama Hospital (Yokohama), Showa University Fujigaoka Hospital (Yokohama), Kitasato University East Hospital (Sagamihara), Kitasato University School of Medicine (Sagamihara), Sagamihara Kyodo Hospital (Sagamihara), Saiseikai Yokohama Tobu Hospital (Yokohama), Yokohama City Minato Red Cross Hospital (Yokohama), Yokohama City University Medical Center (Yokohama), Saiseikai Yokohamasi Nanbu Hospital (Yokohama), Yokohama City University (Yokohama), Yokohama Minami Kyosai Hospital (Yokohama), St. Marianna University, School of Medicine, Yokohama Seibu Hospital (Yokohama), Kanagawa Cancer Center (Yokohama), International Goodwil Hospital (Yokohama), National Hospital Organization Yokohama Medical Center (Yokohama), Yokohama sakae kyosai hospital (Yokohama), Ofuna Chuo Hospital (Kamakura), Shonan Kamakura General Hospital (Kamakura), Fujisawa Shounandai Hospital (Fujisawa), Hiratsuka Kyosai Hospital (Hiratsuka), Hiratsuka City Hospital (Hiratsuka), Kanagawa Prefectural Ashigarakami Hospital (Ashigarakami), Tokai University (Isehara), Niigata Prefectural Central Hospital (Jouetsu), Joetsu General Hospital (Jouetsu), Saiseikai Niigata Daini Hospital (Niigata), Niigata City General Hospital (Niigata), Niigata University Medical & Dental Hospital (Niigata), Niigata Cancer Center Hospital (Niigata), Niigata Prefectural Shibata Hospital (Shibata), Murakami general hospital (Murakami), Niigata Prefectural Yoshida Hospital (Tsubame), Toyama Prefectural central Hospital (Toyama), Koseiren Takaoka Hospital (Takaoka), Kanazawa Medical University (Kahoku), Kanazawa University Hospital (Kahoku), Ishikawa Prefectural Central Hospital (Kanazawa), National Hospital Organization Kanazawa Medical Center (Kanazawa), Kanazawa Redcross Hospital (Kanazawa), University of Fukui Hospital (Yoshida), National Hospital Organization Fukui National Hospital (Tsuruga), Fukui Red Cross Hospital (Fukui), FukuiKen Saiseikai Hospital (Fukui), University of Yamanashi Hospital (Chuo), Nagano Red Cross Hospital (Nagano), Nagano Municipal Hospital (Nagano), Aizawa Hospital (Matsumoto), Shinshu University Hospital (Matsumoto), Iida Municipal Hospital (Iida), Gifu Municipal Hospital (Gifu), Gifu Prefectural General Medical Center (Gifu), Gifu University Hospital (Gifu), Ogaki Municipal Hospital (Ogaki), Gifu Prefectural Tajimi Hospital (Tajimi), Numazu City Hospital (Numazu), Juntendo Shizuoka Hospital (Izunokuni), Shizuoka Cancer Center Hospital (Sunto), Fujinomiya City General Hospital (Fujinomiya), Shizuoka General Hospital (Shizuoka), Shizuoka Kosei Hospital (Shizuoka), Shizuoka City Shizuoka Hospital (Shizuoka), Sizuoka City Shimizu Hospital (Shizuoka), Seirei Hamamatsu General Hospital (Hamamatsu), Hamamatsu University School of Medicine (Hamamatsu), Hamamatsu Medical Center (Hamamatsu), Seirei Mikatahara General Hospital (Hamamatsu), Iwata City Hospital (Iwata), Toyohashi Municipal Hospital (Toyohashi), Aichi Cancer Center Aichi Hospital (Okazaki), Okazaki City Hospital (Okazaki), Anjo Kosei Hospital (Anjo), Nagoya Kyoritsu Hospital (Nagoya), Fujita Health University banbuntane hotokukai hospital (Nagoya), Chubu Rosai Hospital (Nagoya), Social Insurance Chukyo Hospital (Nagoya), Nagoya City Midori Municipal Hospital (Nagoya), National Hospital Organization Nagoya Medical Center (Nagoya), Yokoyama Gastrointestinal Hospital (Nagoya), Aichi Cancer Center (Nagoya), Nagoya University Hospital (Nagoya), Nagoya University Graduate School of Medicine, Gastroenterological Surgery (Nagoya), Nagoya Daini Red Cross Hospital (Nagoya), Nagoya City University　Graduate School of Medical Sciences (Nagoya), Toyota kosei Hoapital (Toyota), Fujita Health University Hospital (Toyoake), Handa City Hospital (Handa), Chita City Hospital (Chita), Aichi Medical University (Aichi), Tosei General Hospital (Seto), Ichinomiya Municlpal Hospital (Ichinomiya), Kainan kosei Hoapital (Yatomi), Mie University (Tsu), SAISEIKAI MATSUSAKA GENERAL HOSPITAL (Matsusaka), Yamada Red Cross Hospital (Ise), Japanese Red Cross Otsu Hodpital (Otsu), Otsu Municlpal Hospital (Otsu), Shiga University of Medical Science (Otsu), Shiga Medical Center for Adults (Moriyama), Nagahama red cross Hospital (Nagahama), Kyoto Second Red Cross Hospital (Kyoto), Nishijin Hospital (Kyoto), Kyoto Prefectural University of Medicine (Kyoto), Japanese Red Cross Kyoto Daiichi Hospital (Kyoto), Kyoto University Graduate School of Medicine (Kyoto), National Hospital Organization kyoto Medical Center (Kyoto), Sumitomo Hospital (Osaka), Osaka North Japan Post Hospital (Osaka), Osaka City Juso Hospital (Osaka), Yodogawa Christian Hospital (Osaka), Osaka Saiseikai Noe Hospital (Osaka), Osaka Medical Center for Cancer and Cardiovascular Diseases (Osaka), Osaka Police Hospital (Osaka), Osaka Red Cross Hospital (Osaka), NTT West Osaka Hospital (Osaka), Osaka City University (Osaka), Nissay Hospital (Osaka), Osaka Koseinenkin Hospital (Osaka), Osaka General Medical Center (Osaka), Suita Municipal Hospital (Suita), Osaka Medical College Hospital (Takatsuki), Kansai Medical University Takii Hospital (Moriguchi), Hoshigaoka Koseinenkin Hospital (Hirakata), Higashiosaka City General Hospital (Higashiosaka), Saiseikai Tondabayashi Hospital (Tondabayashi), National Hospital Organization Osaka Minami Medical Center (Kawachinagano), Kinki University Hospital (Osakasayama), Osaka Rosai Hospital (Sakai), Izumiotsu Municipal Hospital (Izumiotsu), Kishiwada City Hospital (Kishiwada), Kishiwada Tokushukai Hospital (Kishiwada), Rinku General Medical Center (Izumisano), Bell Land General Hospital (Sakai), Kobe City Medical Center General Hospital (Kobe), Kobe Rosai Hospital (Kobe), The Medical Treatment Corporate Corporation Shinko-Kai Association Shinko Hospital (Kobe), Shakaihoken Kobe Central Hospital (Kobe), Nishikobe Medical Center (Kobe), Kobe City Medical Center West Hospital (Kobe), National Hospital Organization Kobe Medical Center (Kobe), Sano Hospital (Kobe), Hyogo Prefectural Awaji Hospital (Sumoto), Kansai Rosai Hospital (Amagasaki), Hyogo Prefectural Nishinomiya Hospital (Nishinomiya), Nishinomiya Municipal Central Hospital (Nishinomiya), Hyogo College of Medicine (Nishinomiya), Kinki Central Hospital of Mutual Aid Association for Public School Teachers (Itami), Himeji St.Mary's Hospital (Himeji), Itami City Hospital (Itami), Takarazuka Municipal Hospital (Takarazuka), Kawanishi City Hospital (Kawanishi), Hyogo Cancer Center (Akashi), Kakogawa West City Hospital (Kakogawa), Nara Prefectural Nara Hospital (Nara), Tenri Hospital (Tenri), Nara Medical University Hospital (Kashihara), Dongo Hospital (Yamatotakada), Japanese Red Cross Wakayama Medical Center (Wakayama), Social Insurance Kinan Hospital (Tanabe), Hashimoto Municipal Hospital (Hashimoto), Tottori Prefectural Central Hospital (Tottori), Tottori Red Cross Hospital (Tottori), Tottori University Hospital (Yonago), National Hospital Organization Yonago Medical Center (Yonago), Matsue Red Cross hospital (Matsue), Shimane University Hospital (Izumo), Shimane Prefectural Central Hospital (Izumo), Okayama University Hospital (Okayama), Kawasaki Medical School Hospital (Kurashiki), Okayama Rosai Hospital (Okayama), Tsuyama Chuo Hospital (Tsuyama), Kurashiki Central Hospital (Kurashiki), Chugoku Central Hospital (Fukuyama), National Hospital Organization Fukuyama Medical Center (Fukuyama), Miyoshi Central Hospital (Miyoshi), Hiroshima City Hospital (Hiroshima), Hiroshima Red Cross Hospital & Atomic-bomb Survivors Hospital (Hiroshima), Hiroshima Prefectural Hospital (Hiroshima), National Hospital Organization Kure Medical Center and Chugoku Cancer Center (Kure), National Hospital Organization Higashihiroshima Medical Center (Higashihiroshima), Ogori Dai-ichi General Hospital (Ymamaguchi), UBE INDUSTRIES,LTD. Central Hospital (Ube), Yamaguchi University Graduate School of Medicine (Ube), Nagato General Hospital (Nagato), Tokushima University Hospital (Tokushima), Tokushima Prefectural Central Hospital (Tokushima), Oe kyodou Hospital (Yoshinogawa), Tokushima Prefectural Miyoshi Hospital (Miyoshi), Anankyoei Hospital (Anan), Kagawa Prefectural Central Hospital (Takamatsu), Kagawa University Hospital (Kita), Kagawa Rosai Hospital (Marugawa), Mitoyo General Hospital (Kanonji), Ehime Prefectural Central Hospital (Matsuyama), Matsuyama Red Cross Hospital (Matsuyama), National Hospital Organization Shikoku Cancer Center (Matsuyama), Ehime University Hospital (Toon), Ehime University Hospital (Toon), Saiseikai Saijo Hospital (Saijyo), Uwajima City Hospital (Uwajima), National Hospital Organization Kochi National Hospital (Kochi), Kochi Health Sciences Center (Kochi), Kochi Medical School Hospital (Nankoku), Kitakyushu Municipal Medical Center (Kitakyushu), Nippon Steel Yawata Memorial Hospital (Kitakyushu), Fukuoka Shin Mizumaki Hospital (Onga), University of Occupational and Environmental Health (Kitakyushu), Saiseikai Fukuoka General Hospital (Fukuoka), Hamanomachi Hospital (Fukuoka), National Hospital Organization Kyushu Medical Center (Fukuoka), Kyushu Cancer Center (Fukuoka), Fukuoka Higashi Medical Center (Koga), Kyushu University Hospital (Fukuoka), Kyushu University Faculty of Medicine (Fukuoka), Fukuoka University Hospital (Fukuoka), Fukuoka Dental College Medical and Dental Hospital (Fukuoka), Japanese Red Cross Fukuoka Hospital (Fukuoka), Kyushu Central Hospital of the Mutual Aid Association of Public School Teachers (Fukuoka), Fukuoka University Chikushi Hospital (Chikushino), Social Insurance Tagawa Hospital (Tagawa ), Kurume University (Kurume), St.Mary's Hospital (Kurume), Omuta City Hospital (Omuta), Kurume University Medical Center (Kurume), Kurume Coloproctology Center (Kurume), Saga Prefectural Hospital Koseikan (Saga), Nagasaki Municipal Hospital (Nagasaki), Nagasaki University Hospital,Division of Surgical Oncology (Nagasaki), Nagasaki University Hospital,Department of Surgery (Nagasaki), Japanese Red Cross Nagasaki Genbaku Hospital (Nagasaki), Oomura City Municipal Hospital (Oomura), National Hospital Organization Nagasaki Medical Center (Oomura), Sasebo Municipal General Hospital (Sasebo), National Hospital Organization Kumamoto Medical Center (Kumamoto), Kumamoto Chiiki Medical Center (Kumamoto), Kumamoto University (Kumamoto), Saiseikai Kumamoto Hospital (Kumamoto), Japanese Red Cross Kumamoto Hospital (Kumamoto), Takano Hospital (Kumamoto), Kumamoto Chuo Hospital (Kumamoto), Yatsushiro Social Insurance General Hospital (Yatsushiro), Health Insurance Hitoyoshi General Hospital (Hitoyoshi), National Hospital Organization Oita Medical Center (Oita), Oita University (Yufu), Kagoshima University Medical And Dental Hospital (Kagoshima), Imamura Hospital (Kagoshima), Nanpuh Hospital (Kagoshima), Saiseikai Sendai Hospital (Satsumasendai), Kirishima Medical Ceter (Kirishima), Okinawa Red Cross Hospital (Naha), Tomishiro Central Hospital (Tomigusuku), Nakagami Hospital (Okinawa),
